# Anterior chamber and retropupillary iris-claw intar ocular lens fixation comparison of clinical outcomes: a systematic review

**DOI:** 10.1097/MS9.0000000000002856

**Published:** 2025-01-09

**Authors:** Khaled Moghib, Izere Salomon, Yasmeen Abdelglel, Samr A. Amer, Ammar Salah, Suhel F. Batarseh, Nagham Bushara Abbas, Amr K. Hassan

**Affiliations:** a Faculty of Medicine, Cairo University, Cairo, Egypt; b Medical research group of Egypt, Negida Academy, Arlington, Massachusetts; c University of Rwanda College of Medicine and Health Sciences, Kigali, Rwanda; d Royal College of General Practitioners, London, United Kingdom; e Faculty of Medicine, Zagazig University, Zagazig, Egypt; f Department of family medicine, Faculty of Medicine, Zagazig University, Zagazig, Egypt; g Faculty of Medicine, Al_Azhar Asuit, Egypt; h Faculty of Medicine, Jordan University of Science and Technology, Irbid, Jordan; i Department of Ophthalmology, Gavin Herbert Eye Institute, University of California, Irvine, California

**Keywords:** anterior chamber IOL, cataract, retropupillary IOL, systematic reviews

## Abstract

**Background:**

In cataract surgery, optimal intraocular lens (IOL) placement is typically within the capsular bag. However, in the absence of sufficient capsular support, alternative techniques such as scleral-sutured IOLs, anterior chamber IOLs, and iris-fixated IOLs either in the anterior chamber or retropupillary are employed. The choice between these methods depends on factors like surgeon expertise, patient-specific anatomy, and clinical circumstances, with anterior chamber iris-claw IOLs offering a more straightforward approach and retropupillary techniques potentially providing additional benefits requiring advanced skills.

**Objective:**

This systematic review aims to evaluate the clinical outcomes of anterior chamber versus retropupillary iris-claw IOLs fixation.

**Methods:**

Adhering to the Preferred Reporting Items for Systematic Review and Meta-analysis guidelines, a comprehensive literature search was conducted using PubMed, Cochrane Central, Scopus, and Web of Science databases until May 2024. These searches focused on specific terms related to the methods, and data were extracted using a standardized online sheet.

**Results:**

The review encompassed nine studies, including two randomized control trials (RCTs) and seven observational studies (Cohort and case controls). A total of 714 patients were evaluated, comprising 295 females (41.3%) and 419 males (58.7%), with a mean age of 60.11 years (SD = 10.22).

**Conclusion:**

The studies underscore the ongoing debate regarding the optimal surgical approach for IOL implantation in cases of insufficient capsular support. Both anterior chamber and retropupillary techniques demonstrates efficacy, with the latter offering potential advantages through retropupillary iris-claw IOL implantation via scleral approach. However, larger-scale studies with longer follow-up periods are still needed to definitively establish the relative merits of the different procedures.

## Introduction

During cataract surgery, the ideal placement of the intraocular lens (IOL) is within the capsular bag, as it closely mimics the natural position of the crystalline lens. However, there are various scenarios where the capsular support may be inadequate for in-the-bag IOL implantation. These situations can arise due to traumatic lens subluxation, complicated cataract extraction, IOL dislocation, zonular dehiscence, or following early congenital cataract removal. Several alternative IOL fixation methods have been developed to address these challenging cases. These include scleral-sutured IOL fixation, anterior chamber IOL placement, and iris-fixated IOL implantation which can be further subdivided into two main techniques: anterior chamber and retropupillary iris-claw IOL^[1-5]^. The choice depends on factors like surgeon experience, patient anatomy, and clinical scenario, with the anterior chamber iris-claw IOL being more straightforward, while the retropupillary approach may offer benefits but require more advanced skills. Regarding the complications of secondary IOL techniques, previous studies have reported the following: angle-supported anterior chamber IOLs have been associated with a higher incidence of bullous keratopathy, lens dislocation, secondary glaucoma, macular edema, and retinal detachment[6]. Scleral-sutured IOLs, while offering the advantage of posterior chamber fixation, involve a more technically demanding surgical procedure with a longer operative time. Inadequate scleral suture fixation can lead to lens tilt, suprachoroidal/vitreous hemorrhage, or retinal detachment. Additionally, conjunctival erosion with suture exposure increases the risk of endophthalmitis, and suture breakage may result in IOL dislocation[7]. In contrast, iris-claw aphakic IOLs are considered by many surgeons as the preferred choice for secondary implantation in adult patients. Since their introduction in the 1960s and 1970s, the iris-claw IOL design has evolved, and its implantation is now viewed as an effective, predictable, and safe option for eyes lacking capsular support. Compared to other secondary IOL techniques, iris-claw implantation is associated with quicker visual recovery, better visual outcomes, and fewer complications. Furthermore, the procedure can be performed with lower invasiveness and in a shorter surgical time. While both anterior and posterior chamber iris-claw IOL implantation have been successfully performed, there is no universal consensus on the optimal placement of these lenses. In this systematic review, we aimed to assess the clinical outcomes between these two procedures.

## Methodology

This systematic review and meta-analysis were conducted following the Preferred Reporting Items for Systematic Reviews and Meta-Analyses (PRISMA) guidelines to ensure rigor and transparency.

### Protocol and registration

Before conducting the review to promote transparency and reduce potential duplication, this protocol has been prospectively registered with PROSPERO, the international database of prospectively registered systematic reviews in health and social care, and the registration number is [CRD42023454029].

The protocol for this systematic review and meta-analysis was meticulously crafted following the PRISMA-P (Preferred Reporting Items for Systematic review and Meta-Analysis Protocols) guidelines to ensure a structured and comprehensive approach^[8,9]^. The updated PRISMA statement coupled with the checklist was used to report this review, as outlined in the Cochrane Handbook[10].

### Study design and search strategy

A comprehensive literature search was conducted using several electronic databases, including PubMed, Cochrane central, Scopus, and Web of Science. The search was completed up to May 2024 and employed specific keywords and Medical Subject Headings terms related to “*Anterior chamber*,” “*Retropupillary*,” “*iris claw intraocular lens*,” and “*cataract surgery*.” The search strategy was designed to capture all relevant studies, and the search terms were redefined iteratively with the assistance of a medical librarian to ensure precision and coverage.

### Study selection

Articles were selected based on predefined inclusion and exclusion criteria. We included studies that met the following criteria: cross-sectional, cohort, case-control, trial, report, and series studies published in English. The population included patients of any age who underwent iris-claw IOL implantation. We considered studies involving anterior and retropupillary iris-claw IOLs, focusing on details of surgical techniques, study characteristics, and summaries of discussions.

Excluded studies were noncomparative studies, review articles, case reports, studies not in English as well as those on iris-claw IOL positions other than anterior retropupillary and studies lacking relevant outcome data.

The study selection process was conducted in two stages: first, titles and abstracts were screened, followed by full-text review and retrieval for further assessment of outcomes and follow-up periods of potentially eligible studies. Two independent reviewers conducted the screening process, and disagreements were resolved through consensus or consultation with a third author.

### Data extraction and quality assessment

After the extraction of potential studies, two reviewers independently screened the full-text articles for eligibility. Afterward, the quality assessment of studies was objectively measured using Cochrane Collaboration’s tool to assess the risk of bias. Disagreement between the reviews on the quality of studies was handled through the direct involvement of the primary author in the evidence-based discussion.

Each article was critically appraised for risk of bias, *RCTS* was assessed using the *Robin 1 tool*, which includes random sequence generation (selection bias), allocation concealment (selection bias), blinding of participants and personnel (performance bias), blinding of outcome assessment (detection bias), incomplete outcome data (attrition bias), selective reporting (reporting bias), and other bias, These were graded as high, low, or unclear, and discrepancies were settled through constructive discussion and reaching a consensus. *Observational studies* were assessed using the *Newcastle – Ottawa Quality assessment scale* for case-control studies, which included three domains, the first is selection, which has four check items: Is the case definition adequate? Representativeness of the cases, selection of controls, definition of controls. The second domain is the comparability of cases and controls on the basis of the design or analysis, and the third is exposure, which includes ascertainment of exposure, is the same method of ascertainment for cases and controls? and non-response rate. The assessment was done using a score, of star for every checked domain, and two stars for comparability, the assessment was one of three, good, fair, and poor quality, good quality is: 3 or 4 stars in the selection domain AND 1 or 2 stars in the comparability domain AND 2 or 3 stars in outcome/exposure domain, fair quality is: 2 stars in selection domain AND 1 or 2 stars in comparability domain AND 2 or 3 stars in outcome/exposure domain, poor quality is: 0 or 1 star in selection domain OR 0 stars in comparability domain OR 0 or 1 stars in outcome/exposure domain.

Subsequently, pertinent data elements were extracted from articles that met the inclusion criteria. (1) characteristics of the studies included, (2) characteristics of the population of studies included, (3) risk of bias domains, and (4) details of surgical techniques were extracted through a predesigned data extraction tool.

## Results

### Studies selection

**Figure 1.**
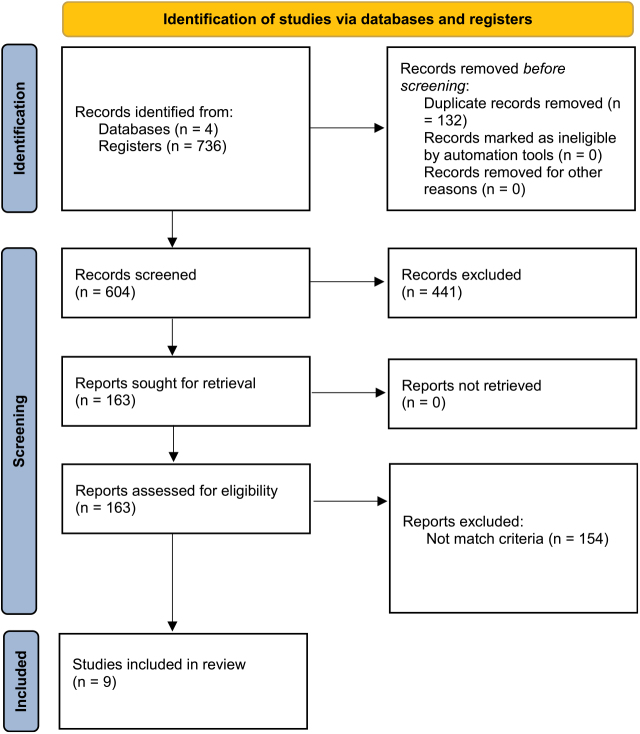
PRISMA flow diagram. **Source**: Page MJ, *et al* BMJ 2021;372:n71. doi: 10.1136/bmj.n71. This work is licensed under CC BY 4.0. To view a copy of this license, visit https://creativecommons.org/licenses/by/4.0/.

Initially, we identified 736 records from four databases which were subsequently screened down to 604 for title and abstract screening after removing 132 duplicate entries. Based on predefined criteria 441 records were excluded. A total of 163 reports were sought for retrieval and were subsequently assessed for eligibility. While 154 full-text articles were excluded during the evaluation process, ultimately 9 studies with 714 patients were included in the review (Fig. 1).

### Characteristics of Included Studies

The review comprised nine studies, including two randomized controlled trials (RCTs)^[5,11]^ and seven observational studies which encompassed a range of cohort and case series designs^[2,12-17]^ (Table 1). A total of 714 patients were included in the analysis, with 295 (41.3%) identified as females and 419 (58.7%) identified as males. The mean age of participants was 60.11 years, with a standard deviation of 10.22 years, highlighting certain limitations within the data.

**Table 1:** Characteristics of included studies

Author, year	Groups	Country, year	Study design	Length of follow (months)	Outcome measurement	Main findings	Summary of discussion
**Helvaci *et al*, 2016**[5]	AC-IFIOL	Turkey, 2016	RCT	6	CDVA, IOP, slit-lamp exam, fundus exam	Both anterior chamber and retropupillary implantation of Artisan IOL showed significant improvement in CDVA with similar outcomes in terms of complications.	The discussion section emphasizes conflicting results on the location of Artisan IOL implantation, stresses the importance of long-term follow-up for safety and efficacy assessment, and calls for more extensive studies with larger sample sizes.
	RP-IFIOL						
**Al-Dwairi *et al*, 2022**[2]	AC-IFIOL	Jordan, 2022	Retrospective cohort	12	Visual acuity	- Patients with retropupillary iris-claw IOL had better visual outcomes compared to those with anterior iris-claw IOL.	The study revealed that retropupillary iris-claw IOL may achieve better visual outcomes without significant postoperative complications, suggesting the need for further research with larger sample sizes.
	RP-IFIOL					- Anterior iris-claw IOL patients experienced more high intraocular pressure readings and macular edema.	
						- The study suggests that retropupillary iris-claw IOL may achieve better visual outcomes without significant postoperative complications.	
**Toro *et al*, 2019**[20]	AC-IFIOL	Germany, 2019	Case series	60	CDVA, IOP, slit-lamp exam, fundus exam, CCT, ECC, CMT on OCT	Both anterior and posterior Iris IOL implantation are effective in treating aphakia without sufficient capsule support, improving visual acuity significantly without serious complications at the 5-year follow-up.	The paper discusses the comparison of anterior and posterior Iris IOL implantation for the treatment of aphakia without sufficient capsule support, aiming to evaluate their long-term efficacy and complication rates.
	RP-IFIOL						
**Hazar *et al*, 2013**[13]	AC-IFIOL	Turkey, 2013	Retrospective case series	12	CDVA, IOP, slit-lamp exam, fundus exam, ECC, FA (if needed)	The three secondary IOL implantation procedures (AC-IFIOL, RP-IFIOL, and SF-IFIOL) demonstrated similar visual efficacy and corneal endothelial cell loss, with SF-PCIOL showing higher IOP elevation but similar rates of other complications.	The study concludes that AC-IFIOL, RP-IFIOL, and SF-IFIOL procedures have similar visual efficacy and corneal endothelial cell loss outcomes, with only a significant difference in intraocular pressure elevation in the SF-PCIOL group.
	RP-IFIOL						
**Mora *et al*, 2018**[14]	AC-IFIOL	Italy, 2018	Retrospective cohort	12	CDVA, IOP, slit-lamp exam, fundus exam, ECC, CMT on OCT	Anterior chamber and posterior chamber iris-claw IOL fixations were equally effective and safe for managing aphakia with inadequate capsular support, with comparable improvements in visual acuity and similar final visual outcomes. The BCDVA at 1 month after surgery was the best predictor of the final BCDVA.	The comparison between anterior and posterior iris-claw IOL fixations showed similar effectiveness and safety in managing aphakia with inadequate capsular support, with comparable visual outcomes, ECD loss, and postoperative complications between the two groups.
	RP-IFIOL						
**Touriño Peralba *et al*, 2018**[15]	AC-IFIOL	Spain, 2018	Retrospective cohort	Median 12	CDVA, IOP, slit-lamp exam, fundus exam, corneal astigmatism, ECC, CMT on OCT	Iris-claw IOL fixation is effective with fewer complications, but prepupillary implantation may lead to more endothelial cell loss and earlier CME onset compared to retropupillary implantation.	The discussion section emphasizes the effectiveness of iris-claw IOL fixation in patients without capsular support, discusses the outcomes of prepupillary and retropupillary implantations, and highlights the need to determine the superior location for long-term results.
	RP-IFIOL						
**Durmus *et al*, 2021**[16]	AC-IFIOL	Turkey, 2021	Retrospective cohort	9	CDVA, endothelial cell loss	- Both prepupillary and retropupillary implantations of iris-claw IOLs led to a significant increase in CDVA.	The study concludes that both prepupillary and retropupillary implantations of iris-claw IOLs resulted in good visual outcomes, with similar rates of endothelial loss and a trend toward reduced complications in the retropupillary group.
	RP-IFIOL					- There was no significant difference in visual gain and endothelial loss rates between the prepupillary and retropupillary implantation groups.	
						- Retropupillary implantation showed an insignificant trend toward a reduced complication rate compared to prepupillary implantation.	
**(Hirashima *et al*, 2010)**[11]	AC-IFIOL	Brazil, 2010	RCT	12	CVA results at 3, 6, and 12 months, complications, endothelial cell loss, and central retinal thickness.	Both iris-fixated PCIOL and iris-claw ACIOL showed significant improvements in corrected visual acuity at 3, 6, and 12 months postoperatively, with no significant difference in outcomes between the two groups. IOL dislocation tended to occur more frequently in the iris-fixated PCIOL group.	The study compared outcomes of iris-fixated PCIOL and iris-claw ACIOL in Marfan syndrome patients, showing similar improvements in CVA at 3, 6, and 12 months postoperatively, with no significant difference in IOL dislocation rates.
	RP-IFIOL						
**(Hernández Martínez and Almeida González, 2018)**[17]	AC-IFIOL	Spain, 2018	Case series	33	Postoperative uncorrected (UDVA) and corrected (CDVA) distance visual acuities, percentage of patients achieving 20/40 or better visual acuity, mean UDVA and CDVA at 6 months postoperatively, surgically induced astigmatism in different incision groups.	Eyes with a scleral tunnel incision had better refractive and visual results than eyes with a corneal incision. Implanting an iris-claw IOL in a retropupillary position through a scleral tunnel incision is an effective and safe alternative to aphakia without capsule support. The study showed improved uncorrected and corrected distance visual acuities postoperatively, with better outcomes in the scleral tunnel incision group.	Implanting an iris-claw IOL in a retropupillary position through a scleral tunnel incision is an effective and safe alternative for managing aphakia without capsule support, providing better refractive and visual outcomes compared to other techniques.
	RP-IFIOL						

### Risk of bias in the included studies

Across nine studies, two were RCTs, seven were retrospective case-control studies. For RCTs, we used ROBIN 1 tool to assess RCTs, and for retrospective case-control studies, we used Newcastle – Ottawa Quality assessment scale for case-control studies.

**Figure 2.**
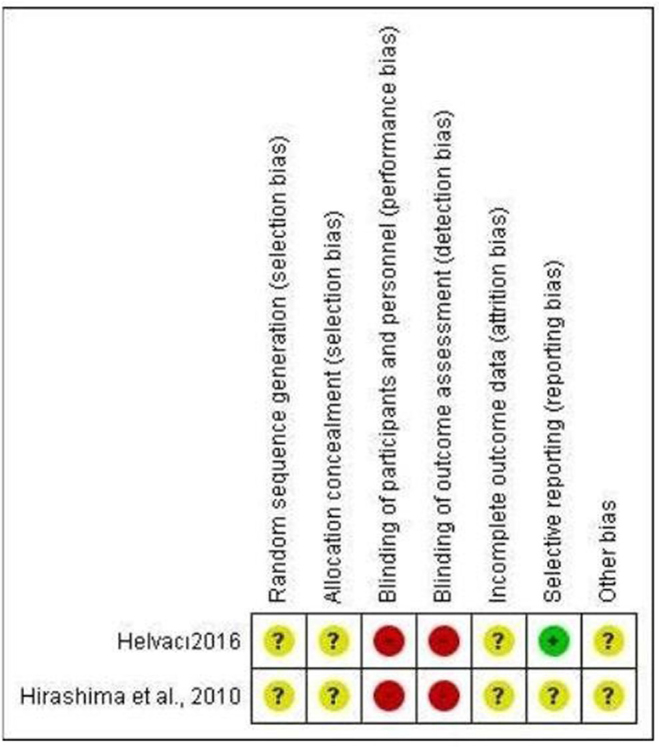
Risk of bias summary for RCTs.

For RCTs, a summary of the risk of bias is shown in Fig. 2. The risk of bias is generally unknown for the two studies^[5,11]^. However, there are some concerns about randomization, allocation and concealment (selection bias) and reporting bias in both studies. We also have some concerns about reporting bias[11].

For our retrospective case-control studies, the Newcastle – Ottawa Quality assessment scale is shown in Table 2. The overall quality is generally good (Good 8^2,^[12]; Good 7^[13-16]^). In Durmus *et al*, the patient was small, which may lead to selection bias[16]. Overall, there are some concerns about the methods of selection of the case and control patients (selection bias).

**Table 2:** Newcastle – Ottawa Quality assessment scale for assessment of Case control studies

Author, Year	Selection				Comparability	Exposure			Overall score
	Is the case definition adequate	Representativeness of the cases	Selection of Controls	Definition of Controls	Comparability of cases and controls on the basis of the design or analysis	Assessment of outcome	Same method of ascertainment for cases and controls	Non-Response rate	Overall score
**Al-Dwairi, 2022**^2^	^*^	^*^	^*^	^*^	^**^	^*^	^*^		8 (Good)
**Durmus *et al*, 2021**^16^	^*^	^*^	^*^	^*^	^**^	^*^			7 (Good)
**Hazar *et al*, 2013**^13^	^*^	^*^	^*^	^*^	^**^	^*^			7 (Good)
**Mora, 2018**[14]	^*^	^*^	^*^	^*^	^**^	^*^			7 (Good)
**Toro *et al*, 2019**^20^	^*^	^*^	^*^	^*^	^**^	^*^	^*^		8 (Good)
**Hernández-Martínez *et al*, 2018**^17^	^*^	^*^	^*^	^*^	^*^	^*^	^*^		7(Good)
**Tourino Peralba *et al*, 2018**^15^	^*^	^*^	^*^	^*^	^**^	^*^			7 (Good)

## Discussion

The choice between the anterior chamber and retropupillary iris-claw IOL implantation involves weighing their respective advantages and disadvantages. While previous studies have examined the surgical procedures (Table 3) and outcomes of these two techniques, the results and conclusions remain contentious.

**Table 3:** Procedures Characteristics

Author, year	Type iris claw	Surgical technique in iris-claw fixation	Main wound location	Wound closure	Instrument for iris fixation	Vitrectomy	Postoperative complications (other complications; number of patients with other complication)
**Helvacı *et al*, 2016**^5^	Artisan	In consideration of our first and preliminary results, both anterior and retropupillary implantation of Artisan IOL are easy applicable surgery procedures	Not mentioned: Anterior chamber Retropupillary:	Interrupted sutures	Enclavation needle	Anterior vitrectomy or PPV for cases with IOL luxation, lens luxation, luxated nigra cataract	Complicated cataract surgery resulted in aphakia
**Al-Dwairi *et al*, 2022**^2^	Artisan	Use of an enclavation needle/microspatula to tuck the iris tissue into the haptics of the IOL	A 5.5-mm corneal incision was made at 12 o’clock	Interrupted 10–0 nylon	Long micro-spatula	Vitrectomy was done in many cases. IOL exchange was performed for patients with anterior chamber IOL or for dislocated IOL.	The most common complication of anterior chamber IOL decentration, high IOP and prolonged use post-operative antiglaucoma
**Toro *et al*,2019**^20^	Artisan	The Artisan aphakia IOL (Ophtec BV, Groningen, The Netherlands) having polymethyl methacrylate IOL with 8.5-mm length	Not mentioned: Cornea	Interrupted 10-0 nylons	Enclavation microspatula	Anterior vitrectomy as required	The most common complication of angle-supported anterior chamber IOLs was bullous keratopathy, followed by lens dislocation, secondary glaucoma, macular edema, and retinal detachment
**Hazar *et al*,2013**^13^	Artisan or Verisyse	A superior 6.0-mm clear corneal incision was performed at the 12-o’clock position. The anterior chamber was filled with a space-maintaining viscoelastic.	At the 12-o’clock position.	Interrupted 10-0 nylon	AC ICIOL: enclavation needle	Anterior vitrectomy if vitreous in AC	Primary early issue was anterior chamber reaction, later cystoid macular edema. Retropupillary iris-fixated IOLs had early anterior chamber reaction, later cystoid macular edema. Scleral-fixated posterior chamber IOLs had early intraocular pressure elevation, later glaucoma. Diverse intraocular pressure elevation across IOL types.
					RP ICIOL: Sinskey hook Or iris spatula		
**Mora *et al*,2018**^14^	Artisan	One haptic positioned behind the iris and enslaved, then the other haptic enclavated	Two side ports were made at the 3 and 9 o’clock positions.	Noncontinuous 10-0 nylon	Enclavation microspatula	Anterior vitrectomy as required or PPV if indicated	AC Cystoid macular edema 33 %
							AC Transiently raised IOP 32%” PC cystoid macular edema 25%
							PC Transiently raised IOP 22%”
**Tourino Peralba *et al*, 2018**	Artisan	Peripheral iris was fixated to the haptics with a needle inserted through the paracenteses	(10 o’clock and 2 o’clock).	Interrupted 10-0 nylon	AC ICIOL: enclavation needle RP ICIOL: reverse Sinskey hook or 27-gauge needle bent 45 degrees	Extensive anterior vitrectomy if no previous peripheral Iridectomy performed previously	Two patients had anterior chamber hemorrhage during surgery, unrelated to IOL implantation. Postoperatively, IOL subluxation occurred in three cases: two in Group 1 and one in Group 2, with onset at around 13.5 and 12 months, respectively. All were successfully reimplanted.
**Durmus *et al*, 2021**^16^	Artisan	Use of an enclavation needle/microspatula to tuck the iris tissue into the haptics of the IOL	2 o’clock and 10 o’clock positions	Non-continuous 10-0 non-absorbable nylon sutures	Enclavation needle	Vitrectomy was performed in cases that had vitreous bands in the pupillary area	The retropupillary group had interop iridodialysis, postop complications of 1 retinal detachment, 1 IOL tilt, and 2 cystoid macular edema.
		For AC fixation, the enclavation was done at the iris midperiphery					
		For retropupillary fixation, the haptics were enclaved one by one behind the iris					
							The prepupillary group had minor 9 o’clock iridodialysis interop, with no significant issues.
**Hirashima *et al*, 2010**^11^	Artisan	MFS: phacoemulsification with an iris-fixated PCIOL or an iris-claw ACIOL implantation.	The haptics were placed at the 3 and 9	Interrupted 10-0 nylon	10.0 polypropylene suture on a long-curved needle o’clock meridians.	N/A	In the PCIOL group, ocular hypertension, iris atrophy, retinal detachment (repaired), IOL capture, and dislocation. Vision outcomes were good after haptic suturing and additional surgeries. In the ACIOL group, ocular hypertension, iris atrophy, retinal detachment (repaired), pupillary block, and pigment deposition.
**Hernández-Martínez *et al*, 2018**^17^	Artisan	IOL was implanted in the usual manner using claw needles or by the Vacufix system	The 5.5-mm main incision was created in the cornea or the scleral tunnel 2.0 mm from the limbus	AC ICIOL: 5 casesscleral incision (Interrupted 10-0 nylon)/23 casescorneal incision (Continued 10-0 nylon) RP ICIOL: 31 casesscleral incision (Interrupted 10-0 nylon)/13 casescorneal incision (Continued 10-0 nylon)	AC ICIOL: 16 casesclaw needle/12 cases-Vacufix system (Ophtec BV)	PPV or anterior vitrectomy through corneal access in eyes that did not require a PPV	There were no complications during surgery. Postoperative complications included choroidal detachment from hypotony, vitreous hemorrhage, retinal detachment after 1-year, ocular hypertension, anterior uveitis, and corneal decompensation requiring keratoplasty after 1 year.
					RP ICIOL: claw needle		

A major concern with AC ICIOL is the potential long-term impact on endothelial cell count (ECC) due to its proximity to the corneal endothelium. Several studies have reported progressive ECC loss, with up to 25% of eyes experiencing more than a 25% reduction in pre-operative ECC over a 3- to 5-year follow-up period[18]. Similarly, ECC loss has been observed with AC ICIOL implantation in aphakic eyes in both adults and children. In contrast, the influence of RP ICIOL on ECC does not appear to be as significant, with some studies reporting no significant change in ECC after a mean follow-up of 5.3 years, while others have shown a stabilization of ECC reduction after an initial decrease of 1 month post-operatively[19].

Helvacı *et al* found that both anterior chamber and retropupillary implantation of Artisan IOLs resulted in significant improvements in corrected distance visual acuity (CDVA), with similar complication rates between the two approaches[5]. The authors emphasized the need for long-term follow-up to fully assess the safety and efficacy of these techniques and called for more extensive studies with larger sample sizes to better understand the relative merits of the different implantation locations[5]. The more recent study by Al-Dwairi *et al* suggests that patients receiving retropupillary iris-claw IOLs may experience better visual outcomes compared to those with anterior iris-claw IOLs[2]. The anterior iris-claw IOL group also had more instances of elevated intraocular pressure and macular edema. These findings indicate that the retropupillary approach may offer advantages in terms of visual outcomes without a significant increase in postoperative complications, though the authors note that further research with larger sample sizes is still needed[2]. Toro *et al* reported that both anterior and posterior iris-fixated IOL implantation were effective in treating aphakia without sufficient capsule support, leading to significant visual acuity improvements and low complication rates over a 5-year follow-up period. The study aimed to directly compare the long-term efficacy and safety of these two implantation techniques[20]. Hazar *et al* compared three secondary IOL implantation procedures – anterior chamber iris-fixated IOLs (AC-IFIOL), retropupillary iris-fixated IOLs (RP-IFIOL), and scleral-fixated posterior chamber IOLs (SF-PCIOL). The authors found similar visual outcomes and corneal endothelial cell loss across the three groups, with the only significant difference being a higher rate of intraocular pressure elevation in the SF-PCIOL group[13]. Mora *et al* found that both anterior chamber and posterior chamber iris-claw IOL fixation were equally effective and safe for managing aphakia with inadequate capsular support. The two approaches yielded comparable improvements in visual acuity and similar final visual outcomes. The authors noted that the best-corrected distance visual acuity (BCDVA) at 1 month after surgery was the best predictor of the final BCDVA[14]. Tourino Peralba *et al* also examined iris-claw IOL fixation, highlighting its effectiveness with fewer complications compared to other techniques. However, the authors noted that prepupillary implantation may lead to more ECC and earlier cystoid macular edema (CME) onset compared to retropupillary implantation. The discussion emphasizes the need to determine the superior location for long-term results[15]. Durmus *et al* similarly found that both prepupillary and retropupillary implantation of iris-claw IOLs led to significant improvements in CDVA[16]. There were no significant differences in visual gain or endothelial loss rates between the two groups, though the retropupillary approach showed an insignificant trend toward reduced complications[16]. Hirashima *et al* compared the outcomes of iris-fixated posterior chamber IOLs and iris-claw anterior chamber IOLs in Marfan syndrome patients[11]. Both approaches resulted in significant improvements in corrected visual acuity at 3, 6, and 12 months postoperatively, with no significant difference in outcomes between the two groups. However, IOL dislocation tended to occur more frequently in the iris-fixated posterior chamber IOL group[11]. Finally, Hernández-Martínez *et al* found that eyes receiving an iris-claw IOL in a retropupillary position through a scleral tunnel incision had better refractive and visual results than those with a corneal incision[17]. This technique was shown to be an effective and safe alternative for managing aphakia without capsule support, providing superior outcomes compared to other approaches[17].

## Conclusion

In conclusion, when the capsular support is inadequate for standard in-the-bag IOL implantation during cataract surgery, several alternative techniques are available, each with its own advantages and disadvantages. Angle-supported anterior chamber IOLs and scleral-sutured IOLs have been associated with a higher risk of complication, such as corneal decompensation, lens dislocation, and retinal issues. In contrast, iris-claw aphakic IOLs are increasingly considered the preferred choice for secondary IOL implantation by many surgeons. This technique offers several benefits, including more predictable and safer outcomes, quicker visual recovery, and lower surgical invasiveness compared to other approaches. While both anterior and posterior chamber iris-claw IOL implantation have been successfully performed, the optimal placement remains an area of ongoing debate and discussion. Ultimately, the choice of technique should be the surgeon’s experience and preference.

## Data Availability

Not applicable.

## Research registration unique identifying number

The study protocol was registered in PROSPERO CRD42023454029.

## References

[1] Agarwal A Jacob S Kumar DA. Handshake technique for glued intrascleral haptic fixation of a posterior chamber intraocular lens. J Cataract Refract Surg 2013;39:317. 23506914 10.1016/j.jcrs.2013.01.019

[2] Al-Dwairi R Saleh O Aleshawi A. Anterior versus retropupillary iris-claw intraocular lens: indications, visual outcome and postoperative complications. Ophthalmol Ther 2022;11:771–84. 35149965 10.1007/s40123-022-00474-2 PMC8927565

[3] Secondary iris-claw anterior chamber lens implantation in patients with aphakia without capsular support. Accessed August 9, 2024. https://pubmed.ncbi.nlm.nih.gov/24489374/10.1136/bjophthalmol-2013-304035 24489374

[4] Simplified and safe method of sutureless intrascleral posterior chamber intraocular lens fixation: y-fixation technique. Accessed August 9, 2024. https://pubmed.ncbi.nlm.nih.gov/24355716/10.1016/j.jcrs.2013.11.003 24355716

[5] Iris-claw intraocular lens implantation: anterior chamber versus retropupillary implantation. Accessed August 9, 2024. https://pubmed.ncbi.nlm.nih.gov/26953023/10.4103/0301-4738.178139 PMC4821121 26953023

[6] The correction of aphakia using anterior chamber intraocular lens. Accessed August 9, 2024. https://pubmed.ncbi.nlm.nih.gov/27815455/10.21873/invivo.10988 27815455

[7] Intraocular lens implantation in the absence of capsular support: a report by the American Academy of Ophthalmology. Accessed August 9, 2024. https://pubmed.ncbi.nlm.nih.gov/12689913/10.1016/s0161-6420(02)02000-6 12689913

[8] Moher D Liberati A Tetzlaff J, PRISMA Group. Preferred reporting items for systematic reviews and meta-analyses: the PRISMA statement. PLoS Med 2009;6:e1000097. 19621072 10.1371/journal.pmed.1000097 PMC2707599

[9] Page MJ McKenzie JE Bossuyt PM. The PRISMA 2020 statement: an updated guideline for reporting systematic reviews. Syst Rev 2021;10:89. 33781348 10.1186/s13643-021-01626-4 PMC8008539

[10] Cochrane handbook for systematic reviews of interventions. Accessed August 9, 2024. https://training.cochrane.org/handbook

[11] Hirashima DE Soriano ES Meirelles RL. Outcomes of iris-claw anterior chamber versus iris-fixated foldable intraocular lens in subluxated lens secondary to marfan syndrome. Ophthalmology 2010;117:1479–85. 20466427 10.1016/j.ophtha.2009.12.043

[12] Toro MD Longo A Avitabile T. Five-year follow-up of secondary iris-claw intraocular lens implantation for the treatment of aphakia: anterior chamber versus retropupillary implantation. PLoS ONE 2019;14:e0214140. 30970023 10.1371/journal.pone.0214140 PMC6457484

[13] Hazar L Kara N Bozkurt E. Intraocular lens implantation procedures in aphakic eyes with insufficient capsular support associated with previous cataract surgery. J Refract Surg 2013;29:685–91. 23898947 10.3928/1081597X-20130723-02

[14] Mora P Calzetti G Favilla S. Comparative analysis of the safety and functional outcomes of anterior versus retropupillary iris-claw IOL fixation. J Ophthalmol 2018;2018:8463569. 30524757 10.1155/2018/8463569 PMC6247566

[15] Touriño Peralba R Lamas-Francis D Sarandeses-Diez T. Iris-claw intraocular lens for aphakia: can location influence the final outcomes? J Cataract Refract Surg 2018;44:818–26. 30055690 10.1016/j.jcrs.2018.05.010

[16] Durmus E Esen F Yenerel M. Clinical outcome and endothelial loss following prepupillary and retropupillary implantation of iris claw intraocular lenses. Int Ophthalmol 2021;41:3961–69. 34324103 10.1007/s10792-021-01965-0

[17] Hernández Martínez A Almeida González CV. Iris-claw intraocular lens implantation: efficiency and safety according to technique. J Cataract Refract Surg 2018;44:1186–91. 30122352 10.1016/j.jcrs.2018.06.049

[18] Galvis V Villamil JF Acuña MF. Long-term endothelial cell loss with the iris-claw intraocular phakic lenses (Artisan®). Graefes Arch Clin Exp Ophthalmol 2019;257:2775–87. 31659458 10.1007/s00417-019-04506-9

[19] Long-term results of correction of high myopia with an iris claw phakic intraocular lens – Abstract. Accessed August 9, 2024. https://europepmc.org/article/MED/10832979 10.3928/1081-597X-20000501-03 10832979

[20] Five-year follow-up of secondary iris-claw intraocular lens implantation for the treatment of aphakia: anterior chamber versus retropupillary implantation. Accessed August 9, 2024. https://pubmed.ncbi.nlm.nih.gov/30970023/10.1371/journal.pone.0214140 PMC6457484 30970023

